# Quantitative Multi-Parametric Magnetic Resonance Imaging of Tumor Response to Photodynamic Therapy

**DOI:** 10.1371/journal.pone.0165759

**Published:** 2016-11-07

**Authors:** Tom J. L. Schreurs, Stefanie J. Hectors, Igor Jacobs, Holger Grüll, Klaas Nicolay, Gustav J. Strijkers

**Affiliations:** 1 Biomedical NMR, Biomedical Engineering, Eindhoven University of Technology, Eindhoven, The Netherlands; 2 Department of Radiology, Translational and Molecular Imaging Institute, Icahn School of Medicine at Mount Sinai, New York, New York, United States of America; 3 Department of Oncology Solutions, Philips Research, Eindhoven, The Netherlands; 4 Biomedical Engineering and Physics, Academic Medical Center, Amsterdam, The Netherlands; Northwestern University Feinberg School of Medicine, UNITED STATES

## Abstract

**Objective:**

The aim of this study was to characterize response to photodynamic therapy (PDT) in a mouse cancer model using a multi-parametric quantitative MRI protocol and to identify MR parameters as potential biomarkers for early assessment of treatment outcome.

**Methods:**

CT26.WT colon carcinoma tumors were grown subcutaneously in the hind limb of BALB/c mice. Therapy consisted of intravenous injection of the photosensitizer Bremachlorin, followed by 10 min laser illumination (200 mW/cm^2^) of the tumor 6 h post injection. MRI at 7 T was performed at baseline, directly after PDT, as well as at 24 h, and 72 h. Tumor relaxation time constants (T_1_ and T_2_) and apparent diffusion coefficient (ADC) were quantified at each time point. Additionally, Gd-DOTA dynamic contrast-enhanced (DCE) MRI was performed to estimate transfer constants (K^trans^) and volume fractions of the extravascular extracellular space (v_e_) using standard Tofts-Kermode tracer kinetic modeling. At the end of the experiment, tumor viability was characterized by histology using NADH-diaphorase staining.

**Results:**

The therapy induced extensive cell death in the tumor and resulted in significant reduction in tumor growth, as compared to untreated controls. Tumor T_1_ and T_2_ relaxation times remained unchanged up to 24 h, but decreased at 72 h after treatment. Tumor ADC values significantly increased at 24 h and 72 h. DCE-MRI derived tracer kinetic parameters displayed an early response to the treatment. Directly after PDT complete vascular shutdown was observed in large parts of the tumors and reduced uptake (decreased K^trans^) in remaining tumor tissue. At 24 h, contrast uptake in most tumors was essentially absent. Out of 5 animals that were monitored for 2 weeks after treatment, 3 had tumor recurrence, in locations that showed strong contrast uptake at 72 h.

**Conclusion:**

DCE-MRI is an effective tool for visualization of vascular effects directly after PDT. Endogenous contrast parameters T_1_, T_2_, and ADC, measured at 24 to 72 h after PDT, are also potential biomarkers for evaluation of therapy outcome.

## Introduction

Photodynamic therapy (PDT) is a photochemistry-based approach for the minimally-invasive local treatment of cancer. It consists of administration of a light-activated chemical, termed a photosensitizer (PS), followed by local irradiation of the tumor with light of the appropriate wavelength, resulting in the generation of cytotoxic species including singlet oxygen [1]. Typically, the light that is used for PDT is in the 600–800 nm wavelength range. PDT is clinically approved and routinely used for treatment of premalignant and malignant non-melanoma skin tumors [2]. Besides, PDT has shown to be promising in the treatment and palliation of head and neck tumors [3], digestive system tumors (e.g. Barrett’s esophagus [4]), and prostate cancer [5]. A comprehensive review on PDT principles and applications can be found in Agostinis *et al*. [6].

Assessment of the treatment outcome is key to therapeutic success, since incomplete treatment may lead to tumor recurrence. The dose-response relation for PDT is notably complex, meaning that therapeutic outcome can vary despite standardized dose. Moreover, light dose and drug dose need to be distinguished. Light dose is usually defined as the fluence at the tumor surface, while drug dose is defined as the amount of injected photosensitizer. However, the actual quantities of photosensitizer and light fluence may vary within tumors and between subjects, due to biological heterogeneity. As a result, treatment response is difficult to predict, and should therefore be evaluated for each individual subject. Preferably, treatment efficacy should be monitored early after therapy, so that poorly responding tumors can be timely identified, in which case repeated or alternative treatment can be started. Furthermore, treatment evaluation should preferably be imaging-based, to enable distinction of spatial heterogeneities in response, which are inherent to tumor non-uniformity of PDT light and drug doses.

PDT causes direct tumor cell damage resulting in apoptosis or necrosis [7]. Additionally, the treatment might lead to vascular occlusion, cutting off the vital supply of oxygen and nutrients to the tumor [8]. Magnetic resonance imaging (MRI) offers techniques to depict both mechanisms of action in a non-invasive and longitudinal fashion. Conventional MRI has been used for PDT response evaluation in a couple of studies, mostly employing T_2_- and diffusion-weighted imaging to identify qualitative signal changes or alterations in gross anatomy [9–12]. Contrast-enhanced (CE) MRI has shown promise for measuring vascular effects of PDT in mammary adenocarcinoma [13] and prostate tumors [11]. The water apparent diffusion coefficient (ADC) is a well-known biomarker to evaluate treatment response in oncology [14]. The ADC is sensitive to necrosis and apoptosis after radiotherapy and chemotherapy [15]. Nevertheless, quantitative MRI has been applied in a few studies only to study PDT response [16–18]. Most of these studies focused on a single or a small set of contrast parameters, whereas the spectrum of biological effects associated with PDT calls for a multi-parametric imaging approach. Besides, combined multi-parametric information can often improve accuracy of treatment evaluation by MRI [19–21]. We hypothesize that systematic quantitative multi-parametric imaging could help in identifying the most sensitive (combination of) readouts of treatment success.

In view of this hypothesis, the goal of this study was to identify quantitative MRI biomarkers that are suitable for assessment of PDT response. To this end, we evaluated changes in T_1_, T_2_, ADC, and DCE-MRI derived vascular parameters upon PDT in a mouse cancer model up to 72 h after treatment. The CT26.WT colon carcinoma model was used, because this tumor is well-perfused and characterized by little spontaneous necrosis [22], allowing selective detection of treatment-induced vascular effects and tissue necrosis.

## Materials & Methods

All animal experiments were performed according to the Directive 2010/63/EU of the European Parliament and approved by the Animal Care and Use Committee of Maastricht University (protocol: 2012–139). Mice were anesthetized using isoflurane in medical air. At the end of experiments, mice were humanely sacrificed by cervical dislocation under anesthesia.

### Mouse cancer model

CT26.WT murine colon carcinoma cells (American Type Culture Collection (ATCC), CRL-2638) were cultured as a monolayer at 37°C and 5% CO_2_ in RPMI-1640 medium (Invitrogen, Breda, The Netherlands), supplemented with 10% fetal bovine serum (Greiner Bio-One, Alphen a/d Rijn, The Netherlands) and 50 U/ml penicillin/streptomycin (Lonza Bioscience, Basel, Switzerland). Early passages (9–13) of the original ATCC batch were used for inoculation. 10–12 week-old BALB/c mice (Charles River, Maastricht, The Netherlands) were inoculated with 2*10^6^ cells subcutaneously in the right hind limb. Tumors became palpable at 3 to 5 days after inoculation. PDT treatment was performed when tumor diameter reached 10 mm, approximately after 10 days. Average tumor volume was 228 ± 174 mm^3^ (mean ± SD) at the day of treatment.

During photosensitizer injection, PDT, and MR imaging, mice were anesthetized using isoflurane (3.5% for induction, 1–2% for maintenance) in medical air flowing at 0.4 L/min. Breathing rate was monitored with a pressure balloon. During PDT and MRI, body temperature was monitored using a rectal probe, and kept at 36–37°C using a warm water circuit.

### Study design

In total, *n* = 22 PDT treated mice and *n* = 10 untreated control mice were used in this study (**S1 Fig**). All PDT treated tumor mice were scanned the day before and directly following PDT. After PDT, which was performed outside the MRI scanner, it took approximately 30 min to put the animal in the MRI scanner and start the scanning session. The total scanning time for the complete multi-parametric MRI protocol was approximately 2.5 h. Subsequently, one group of *n* = 7 mice was killed for histology. Of the remaining 15 mice, *n* = 10 were scanned at 24 h and killed afterwards, whereas *n* = 5 were scanned at 72 h and then followed for at most 2 weeks, during which tumor size was measured 3 times a week with a caliper. One control group of *n* = 5 untreated tumor mice with equal starting tumor size as the PDT group was scanned on 3 consecutive days. After the last scan, these control mice were killed. A second control group of untreated tumor mice (*n* = 5) with equal starting tumor size as the PDT group was scanned on 2 consecutive days, and at 72 h, and then followed for at most 2 weeks, during which tumor size was measured 3 times a week with a caliper. In all groups, a tumor volume of 1500 mm^3^ was used as a humane endpoint.

### Photodynamic therapy

The photosensitizer Bremachlorin, also known outside the EU as Radachlorin, was kindly supplied by Harrie Vink and Andrei Reshetnickov from Rada-Pharma International B.V. It is a mixture of chlorins in 0.35% (w/v) aqueous solution for intravenous injection, with chlorin e6 as its main constituent. A dose of 20 mg/kg body weight Bremachlorin was administered via the tail vein 6 h before light exposure. The hind limb was shaved, and a mask of black paper was positioned around the tumor to avoid light entering the surrounding tissue. Subsequently the tumor was exposed to 655 nm light, delivered by a laser diode (WSLB-650-002-H, WaveSpectrum, Beijing, China) connected to a fiber, which was terminated by a pair of lenses to form a collimated beam with a diameter adjustable to the size of the tumor. The beam was aimed onto the skin covering the tumor, at an irradiance of 200 mW/cm^2^. The laser was turned on for 10 min, resulting in a total fluence of 120 J/cm^2^. Mice received preventive analgesia (s.c. injection of 0.05 mg/kg buprenorphine) 30 min before PDT, and every 12 h during the first 2 days after PDT.

### MRI protocol

All scans were performed with a 7 T MR scanner (BioSpec 70/30 USR, Bruker) using a quadrature 72-mm-diameter transmit and receive birdcage coil. The tumor-bearing paw was gently fixed in a stretched position. Degassed ultrasound gel was applied onto the tumor as a susceptibility matching medium to reduce magnetic field inhomogeneities at air-tissue interfaces. A T_2_-weighted multi-slice spin echo scan was acquired for anatomical reference, with sufficient axial slices (typically 10–16) to cover the entire tumor. Scan parameters were as follows: TR = 1000 ms, TE = 30 ms, matrix = 128x128, slice thickness = 1.0 mm, slice gap = 0.1 mm, FOV = 4x4 cm^2^. The same geometry was used for T_2_- and ADC-mapping. T_2_ maps were acquired using a T_2_-weighted MLEV-prepared [23] GE-EPI sequence with TR = 2000 ms, NA = 2, and 7 TE values (0.9, 14.5, 28.1, 41.7, 55.3, 68.9, and 82.6 ms). ADC maps were acquired with a double spin-echo prepared diffusion-weighted EPI sequence with TE = 41 ms, TR = 4000 ms, NA = 4, slice thickness = 1.0 mm, slice gap = 0.1 mm, FOV = 4x4 cm^2^, and 4 b-values (0, 100, 200, and 400 s/mm^2^) with diffusion gradients in 3 orthogonal directions.

T_1_-mapping was performed using a 3D FLASH sequence with variable flip angle [24]. Sequence parameters were: TR = 20 ms, TE = 3.2 ms, 7 flip angles (2°, 3°, 5°, 7°, 10°, 13°, and 20°), NA = 2, matrix = 128x128x39, FOV = 40x40x22 mm^3^. DCE-MRI was performed using the same 3D FLASH sequence with fixed flip angle and shorter TR and TE. Sequence parameters were: TR = 3 ms, TE = 1 ms, flip angle = 7^o^, NA = 1, acquisition matrix = 128x69x17 (zero-filled to 128x128x39), FOV = 40x40x22 mm^3^. The DCE-MRI sequence was repeated for 15 min with a temporal resolution of 3.5 s to capture the dynamic influx of contrast agent in the tumor. Dotarem was injected 2 min after start of the scan with a dose of 0.3 mmol Gd/kg b.w. in 5 s using a syringe pump (Fusion 100, Chemyx Inc., Stafford, TX, USA), followed by a saline flush.

Both T_1_-mapping and DCE-MRI scans are sensitive to RF transmit B_1_ deviations and inhomogeneities. Therefore an RF flip angle correction map was acquired with a 3D FLASH sequence using the 180° signal null approach [25]. Sequence parameters were: TR = 200 ms, TE = 3.2 ms, 3 flip angles (145°, 180°, and 215°), NA = 1, matrix = 128x128x39, FOV = 40x40x22 mm^3^.

### MR image analysis

All image analysis was performed using homemade scripts written in Matlab R2014a. Manual segmentation of tumors was performed based on the T_2_-weighted spin echo scans, in which the tumor signal was hyperintense compared to muscle tissue. 3D FLASH acquisitions were down-sampled in the third dimension to spatially match the 2D multi-slice acquisitions.

For flip-angle correction the signal intensity (S) in each voxel as function of the nominal flip angle (FA_n_) was fitted to S = |a*FA_n_ + b|, and a flip-angle correction map ζ was constructed according to ζ = -(a/b)*180°. For the T_1_-mapping and DCE-MRI scans the actual flip angle in each voxel (FA_a_) was obtained by correcting the nominal flip angle using FA_a_ = ζ*FA_n_.

T_2_-maps were reconstructed by fitting signal intensity (S) as function of TE to S~Exp(-TE/T_2_). T_1_-maps were calculated by fitting signal intensity (S) as function of actual flip angle (FA_a_) to S~Sin(FA_a_)*(1-Exp(-TR/T_1_))/(1-Cos(FA_a_)Exp(-TR/T_1_)). ADC values per diffusion-gradient orientation were calculated by fitting signal intensity (S) as function of b-value (b) to S~Exp(-ADC*b). A mean orientation-invariant ADC value was obtained by averaging the ADC values of the different diffusion-directions.

From the pre-contrast longitudinal relaxation rate R_1,pre_ = 1/T_1,pre_ and the dynamic signal changes acquired with a single flip angle during contrast agent influx, the dynamic changes in R_1_(t) = 1/T_1_(t) were calculated. Subsequently, the dynamic changes in contrast agent concentration ([CA]) were calculated using R_1_(t) = R_1,pre_ + r_1_*[CA], where r_1_ = 3.53 mM^-1^s^-1^ is the relaxivity of Dotarem at 7 T [26].

For the DCE-MRI, area under the curve (AUC) of Dotarem concentration as function of time was used as a measure of contrast agent uptake by the tumor. Additionally, the standard Tofts-Kermode (TK) model [27] was applied to estimate K^trans^ (transfer constant describing exchange between the blood plasma and the extravascular extracellular space (EES)) and v_e_ (the volume fraction of the EES). The arterial input function (AIF) was constructed from a bi-exponential function with amplitudes A_1_ = 5.36 mM and A_2_ = 1.27 mM, and time constant τ_1_ = 5.36 s and τ_2_ = 915 s. A_1_ was estimated from the injected dose divided by the average blood volume for BALB/C mice [28], corrected for a hematocrit level of 0.563 (value provided by animal supplier), whereas time constant τ_1_ was taken from literature [29]. A_2_ and τ_2_ were based on previous experiments [22].

### Histological analysis

Immediately after killing the animals, the skin covering the tumor was removed, and tumors were marked with colored lines (MD100-1KT, Sigma Aldrich) as anatomical landmarks. Tumors were excised and snap-frozen in a cold isopentane slush, and then stored at -80°C. For histological analysis, tumors were cut in 8 μm axial sections using a cryomicrotome (Shandon Cryotome, Thermo Fischer Scientific). Sections were stained with a NADH diaphorase tissue viability staining [30] or Hematoxylin and Eosin (H&E).

For the NADH-diaphorase stainging, the cryo-sections were briefly air-dried, and subsequently incubated at 37 °C for 1 h in Gomori-Tris-HCl buffer (pH 7.4) containing β-NAD reduced disodium salt hydrate (Sigma-Aldrich, St. Louis, MO, USA, 0.71 mg/ml buffer solution) and nitro blue tetrazolium (Sigma- Aldrich, 0.29 mg/ml buffer solution). Specimens were then washed and mounted for analysis. Brightfield microscopy images of entire sections were performed by mosaic acquisition at 20x magnification.

### Statistical analysis

Average endogenous contrast parameter values were calculated in the entire tumor. For DCE-MRI, pixels were classified as non-enhanced if the median contrast agent concentration after injection was smaller than twice the standard deviation of the pre-contrast signal intensity. K^trans^ and v_e_ were averaged over tumor pixels that satisfied the following criteria: 1) pixels were enhanced; 2) R^2^ of the TK model fit was larger than 0.80; 3) the maximum concentration was reached before 800 s after bolus injection; 4) K^trans^ and v_e_ values were physiologically realistic (0 ≤ K^trans^ and 0 ≤ v_e_ ≤ 1).

Statistical analyses were performed in SPSS software (version 22). Levene’s test was used to test for equality of variances before comparing means of two groups, and an equal-variance or unequal-variance two-tailed t-test was used accordingly. A *p* value of less than 0.05 was considered statistically significant.

## Results

### Tumor volume

**Fig 1:**
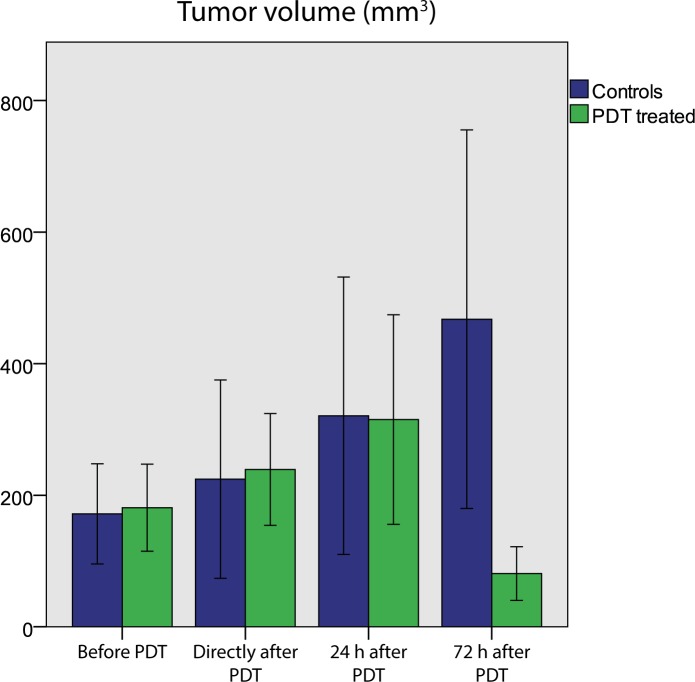
Mean tumor volume of control animals and PDT-treated mice at all time points, calculated by integrating the volumes of all pixels within manually drawn tumor ROIs. At 72 h after PDT, the average volume of treated tumors was significantly different than untreated ones (independent *t*-test, *p* = 0.038). Tumors of treated mice at 72 h after PDT were also significantly smaller than right after PDT (paired *t*-test, *p* = 0.010).

PDT induced visible and palpable effects in the tumor-bearing hind limb. Within a day, there was swelling and darkening of the skin over the tumor. In some animals, a red ring occurred on the skin around the tumor. Two to three days after PDT, a dry necrotic crust had formed. Based on the MRI-based tumor segmentation, the average tumor volume decreased in PDT treated mice after 72 h, while control tumors continued to grow (**Fig 1**). For two of the five mice that were followed for 2 weeks after PDT, there was complete remission, and only a small scab remained at the position of the tumor. In the other 3 mice, there was tumor growth arrest or complete necrosis in the bulk of the tumor, but also continued growth of unsuccessfully treated tumor tissue.

### Endogenous contrast MRI

**Fig 2:**
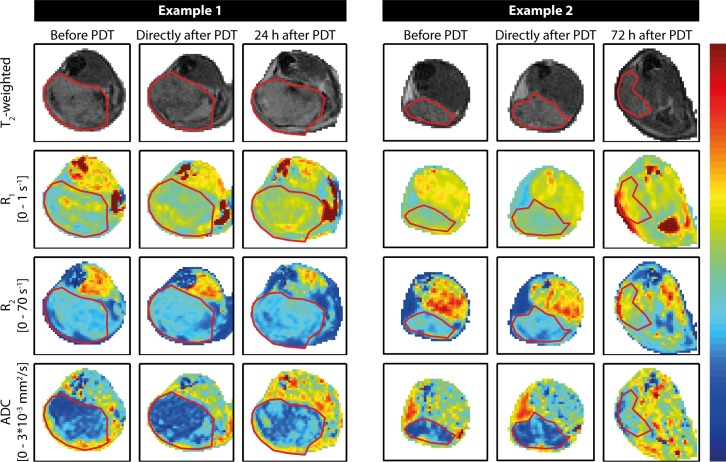
Representative examples of maps of endogenous MR parameters of two mice. First, second, and third columns show measurements of a single mouse obtained before, right after, and 24 h after PDT, respectively. The other three columns contain data acquired before, right after, and at 72 h post PDT of another mouse. The upper row contains T_2_-weighted anatomical reference images, which were used for tumor segmentation, indicated by the red contours. Rows 2 to 4 show R_1_ maps, R_2_ maps, and ADC maps, respectively. The color bar corresponds to the range of values indicated on the left.

The effect of PDT on endogenous MR parameter maps was largely consistent in all mice. **Fig 2**shows representative examples of endogenous parameter maps of two animals, up to 24 h and 72 h after PDT, respectively. R_1_ and R_2_ values in the tumors were rather homogeneous and unaffected by PDT up to 24 h. Typically, widespread increases in tumor R_1_ and R_2_ were visually observed in the maps only as late as 72 h after treatment, although 5 out of 9 animals showed locally increased R_1_ values at 24 h. Tumor ADC values were increased at 24 h and 72 h after treatment. Most mice presented a dry scab of severe necrosis, associated with large R_1_ and R_2_, and low ADC in the superficial tumor regions. Except for low ADC values in such regions with advanced necrosis, the ADC in the bulk of the tumor at 72 h was increased compared to the 24 h time point. Decrease in R_1_ and R_2_, and increase in ADC were observed directly after treatment in the skin covering the tumor and the muscle underneath, which could be attributed to therapy-induced edema.

**Fig 3:**
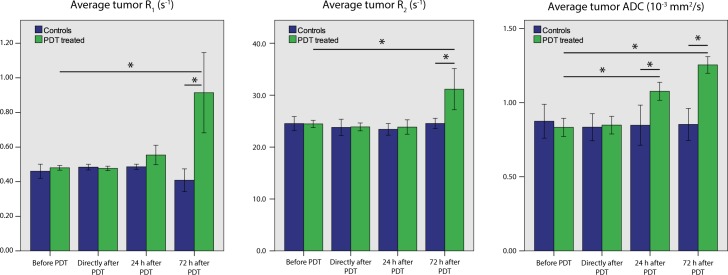
Mean R_1_, R_2_, and ADC values of tumors of non-treated mice and treated mice. At 24 h, ADC values were significantly different for treated versus control mice (independent *t*-test, *p* = 0.002) as well as for treated versus baseline (paired *t*-test, *p* = 0.003). At 72 h, all parameters were significantly increased, compared to control animals at the same time point (independent *t*-test, *p* = 0.009 for R_1_, *p* = 0.010 for R_2_, *p* < 0.001 for ADC) as well as compared to baseline values (paired *t*-test, *p* = 0.019 for R_1_, *p* = 0.027 for R_2_, and *p* < 0.001 for ADC).

The average R_1_, R_2_ and ADC tumor values of treated and untreated mice for the different time points are summarized in **Fig 3**. Average tumor R_1_ and R_2_ were significantly increased at the 72 h time point only. Average tumor ADC was significantly increased at 24 h and 72 h after treatment. R_1_, R_2_, and ADC values remained constant for the control mice.

### Histology

**Fig 4:**
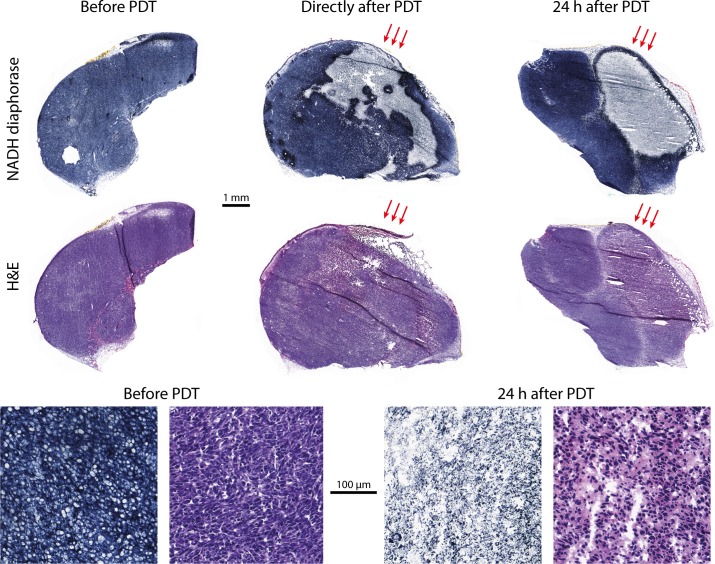
Representative examples of histology sections. NADH diaphorase stained sections (top row) and H&E sections (middle row) of an untreated tumor (left), a tumor directly after PDT (center), and a tumor 24 h post PDT (right). Overall, control tumors were almost completely positively stained, indicating full viability. Directly after PDT, some negative (non-viable) patches were observed. After 24 h, tumors were mostly completely negatively stained, but for the specific section shown here the tumor also contained a large viable region. Red arrows indicate the angle of light incidence during PDT. In the bottom row, close-up images of a viable part in NADH and H&E stained sections are shown on the left. The two close-up images in the bottom right are NADH and H&E stained sections of a non-viable tumor, 24 h after PDT.

Histology revealed changes in tumor tissue viability early after PDT, despite the absence of changes in R_1_, R_2_, and ADC. **Fig 4**shows some representative NADH diaphorase stained sections of tumors excised at different time points. A patchy pattern with viable and non-viable tumor tissue was observed directly after PDT. At 24 h after PDT, all tumors contained mostly non-viable cells. H&E staining confirmed that the regions which did not stain with NADH diaphorase were non viable, showing eosinophilic cytoplasm, hyperchromatic nuclei, and fragmentation of nuclei and cells. Interestingly, a NADH diaphorase viable tumor rim of 50–150 μm width just below the skin was observed in many treated tumors 24h after PDT. Control tumors were mostly completely viable, with the exception of some small necrotic regions.

### DCE-MRI

**Fig 5:**
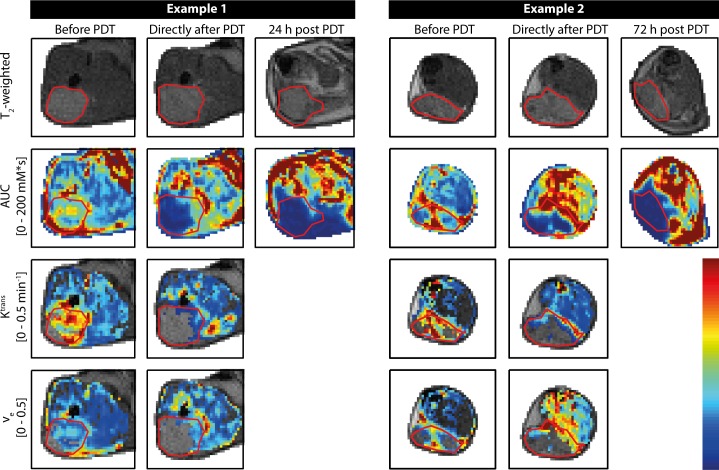
Representative examples of maps of AUC, K^trans^, and v_e_ of two mice. The upper row shows T_2_-weighted anatomical reference images, and the red contours outline the tumors. Example 1 is a mouse measured before, right after, and 24 h after PDT. Example 2 is a mouse measured before, right after, and at 72 h post PDT. For both mice, AUC was significant at baseline in almost the entire tumor. Directly after PDT, AUC in the whole tumor was reduced and the superficial part was non-enhanced (AUC = 0). For K^trans^ and v_e_ only the contrast-enhanced pixels in the tumor are color coded.

In stark contrast to R_1_, R_2_, and ADC, response in DCE-MRI scans was observed directly after PDT (**Fig 5**). Area under the curve (AUC) was used as measure for the degree of contrast agent uptake. Whereas prior to treatment tumor AUC was generally similar or higher as compared to surrounding muscle, the AUC was sharply decreased in large areas of the tumor directly after PDT. The region with low AUC extended to the whole tumor and into the surrounding muscle at 24 h and later. Interestingly, while the tumor AUC was decreased, remote muscle tissue distant from the treatment area became more permeable to contrast agent.

**Fig 6:**
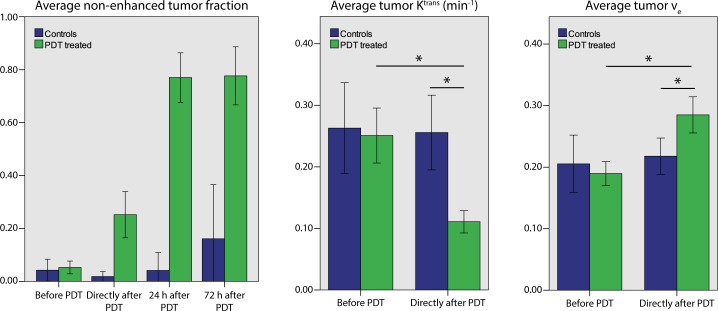
Average tumor values of DCE-MRI derived parameters at different time points before and after PDT, for treated and non-treated animals. Based on independent *t*-tests, average non-enhanced tumor fractions were significantly different between treated and non-treated animals right after, and 24 and 72 h after PDT (all: *p* < 0.0005). For all time points after PDT, the repeated measurements difference of treated animals compared to before PDT was significant (all: *p* < 0.0005), while this was not the case for controls. Directly after PDT, the average tumor K^trans^ decreased and v_e_ increased significantly compared to baseline based on repeated measures (both: *p* < 0.0001), and also compared to untreated animals at the same time point (*p* < 0.0001 and *p* = 0.038, respectively).

**Fig 6**summarizes the non-enhanced tumor fraction and DCE-MRI kinetic parameters for the treatment and control groups. Directly after PDT, the average non-enhanced tumor fraction for treated mice was significantly higher than baseline. It further increased to 77% at 24 h and remained at roughly this value up to 72 h post PDT. K^trans^ in tumor tissue with significant AUC was significantly lower directly after PDT, as compared to baseline values and control tumors. The extracellular extravascular volume fraction v_e_ was significantly higher at this time point. K^trans^ and v_e_ at 24 h and 72 h after PDT are not reported, since these parameters could only be calculated in a small number of tumor pixels at these time points, e.g. due to insignificant AUC.

**Fig 7:**
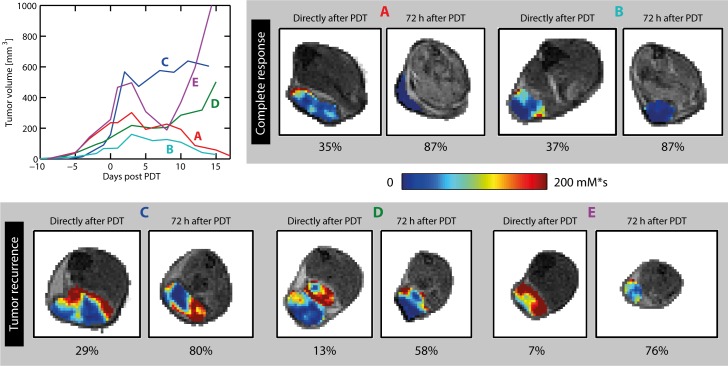
Tumor growth curves and contrast enhancement (AUC) maps of five mice. For two animals (A and B), complete tumor remission was seen after 2 weeks. In the other 3 animals (C, D, and E), tumor growth arrest or volume reduction was seen in the first days after PDT, followed by recurrence of growth. For each animal, AUC maps of a single representative tumor slice are displayed, acquired right after PDT, and 72 h after PDT. The AUC in the tumor is color-coded on top of the grayscale anatomical reference images. At 72 h, no enhancement was observed in the tumors of mice A and B, while significant enhancement was seen in mice C-E. The non-enhanced volume fractions of the entire tumors are listed below the AUC maps.

For the five mice that were followed for up to 2 weeks after PDT, **Fig 7**shows tumor growth curves based on caliper measurements, and AUC maps obtained right after PDT and 72 h after PDT. At both time points, the two animals with complete response (A and B) had higher non-enhanced fractions than the three animals with tumor recurrence (C–E). Interestingly, the location of tumor recurrence corresponded to the region with the highest AUC at 72 h post PDT in animals C–E. For example, in animal C, significant contrast enhancement was only observed in the 3 most distal tumor slices. Indeed, recurrence of tumor growth occurred in the distal tumor zone after 5 days, while the remainder of this tumor turned necrotic. **S2 Fig** shows K^trans^ maps of the entire tumor of 3 animals right after PDT, as well as AUC maps at 72 h post PDT. Regions of tumor recurrence were characterized by significant contrast enhancement directly after PDT, and K^trans^ values above the treated tumor average (*i*.*e*. K^trans^ > 0.11 min^-1^). However, some successfully treated tumor tissue also showed contrast enhancement and high K^trans^ values. None of the animals with tumor recurrence had lower tumor average K^trans^ right after PDT (0.14, 0.20, and 0.10 min^-1^) than those with complete response (0.10 and 0.07 min^-1^).

## Discussion

Multi-parametric MRI was performed to characterize changes in tumor tissue structure and vasculature in response to PDT in a murine tumor model. Our goal was to identify those imaging biomarkers that could be suitable for early evaluation of PDT outcome, preferably within one day after treatment.

DCE-MRI showed the fastest response after treatment. Directly after treatment, considerable tumor parts were non-enhanced, which we attribute to vascular occlusion induced by PDT. In the remaining tumor tissue K^trans^ was decreased compared to baseline and controls, indicating that blood perfusion was compromised. In this tissue the process towards vascular stasis may still have been in progress, as at 24 h and later the complete tumor remained non-enhanced after contrast agent injection. The DCE-MRI data of 5 animals that were sacrificed 2 weeks after PDT suggested that remaining contrast-enhancement at 72 h is an indicator for tumor recurrence. Future work could be aimed at optimizing the PDT to prevent tumor recurrence using DCE-MRI as an early indicator of treatment success.

It is known that PDT can induce severe and quick vascular responses [8]. Vascular occlusion is believed to be initiated by endothelial cell damage, as a result of singlet oxygen production by photosensitizer bound to membranes and other vital parts of endothelial cells. This leads to disintegration of the cytoskeletal structure, cell rounding, and opening of the tight junctions between endothelial cells, exposing the basement membrane. Platelets and leukocytes can bind to these sites of damage, triggering vessel constriction and platelet aggregation through the release of signaling substances. Secondly, direct platelet damage caused by activated photosensitizer in the blood is a mechanism that likewise leads to blood stasis.

Similar observations concerning the vascular effects of PDT using DCE-MRI were done by Zilberstein *et al*. [31], although in their study tumor illumination was started immediately after injection of the photosensitizer. They observed a complete arrest of vascular perfusion 24 h post PDT, but also a marked increase in vessel permeability 1 h post treatment. We did not observe increased K^trans^ directly after PDT, but rather a decrease. This difference might be due to the fact that our first DCE-MRI measurement was performed at approximately 2.5 h instead of 1 h after PDT. At 2.5 h, the vascular damage process may have progressed from vascular rupture towards blood flow stasis.

In contrast to DCE-MRI, the endogenous contrast parameters T_1_, T_2_, and ADC only significantly changed at the later time points. This was somewhat surprising in view of the structural tissue defects that were observed in histological analysis for the treated tumors at this time point. Similarly, Haider *et al*. found unaltered T_2_-weighted contrast in human prostate 1 week after PDT [11]. On the other hand Fei *et al*. reported an increase in T_2_ at 24 h post PDT [32]. In the latter study, a different tumor model, photosensitizer, and treatment protocol were used. Specifically, tumors were exposed to light 48 h after photosensitizer injection, which might have resulted in more cellular uptake than in our study. Wang *et al*. studied ADC changes in response to PDT with a phthalocyanine photosensitizer and observed increased ADC values in tumors 24 h post treatment [17], in line with our findings.

The DCE-MRI enhancement maps revealed considerable volumes of non-perfused muscle around the treated tumors. Muscle damage surrounding the tumor was also observed in H&E sections. This could be caused by ischemia as a result of PDT-induced vascular occlusion. Alternatively, the tumor-to-muscle photosensitizer uptake ratio may have been too low to selectively ablate the tumor. In an HT29 human colorectal xenograft model, photosensitizer fluorescence intensity in tumors was generally less than 2 times higher than in muscle [33].

Generally, PDT is based on a combination of direct cellular damage and vascular shut-down. The relative contributions of both mechanisms depend on the pharmacokinetics of the photosensitizer, but can be manipulated by varying the time between injection and light administration. For our study design, we expected that the direct cellular pathway would be most dominant, as Park *et al*. reported a clearance of Bremachlorin from the blood of C57BL/6 mice: 80% clearance was found 6 hours after i.v. injection [34]. However, our DCE-MRI results suggest that sufficient photosensitizer was present in the blood after 6 h for a strong vascular effect in both tumor and muscle tissue. In order to achieve a more tumor-specific treatment, a longer drug-light interval may be required, to reach sufficient blood clearance and prevent damage to normal tissues through vascular effects.

We have shown in a mouse model that MRI, especially DCE-MRI, is a powerful tool for visualization of tumor response to PDT. Our results also suggested a relation between contrast-enhancement at 72 h post PDT and tumor recurrence, which is crucial for the clinical translatability of the methods. Future research will be aimed at investigating if DCE-MRI measurements early after PDT can be used to classify tumor tissue as successfully treated (viable) or non-successfully treated (non-viable), based on a spatial comparison with histological slice stacks of entire tumors. Furthermore, a study in which more animals are followed-up for a longer period after therapy would allow determining the actual predictive value of the MR biomarkers with regard to long-term therapeutic outcome.

## Conclusion

We presented a multi-parametric MRI follow-up of PDT in a mouse model of cancer. Shut-down of tumor perfusion was observed by DCE-MRI directly after PDT. A late response to the treatment was observed in the endogenous contrast parameters T_1_, T_2_, and ADC. The methods presented here can be applied for optimization of PDT treatment protocols, mechanistic research, but should also be clinically translatable for treatment evaluation of patients.

## Supporting Information

S1 Fig Overview of experiment groups and their timelines. Numbers of animals are indicated per group. Grey blocks represent different measurement days. The following abbreviations are used: **CT26**: subcutaneous inoculation of CT26.WT tumor cells. **MRI**: Multi-parametric MRI scan session (approximate duration: 2.5 h). **PS**: photosensitizer injection, 6 h before PDT. **PDT**: photodynamic therapy (10 min tumor irradiation). **†**: kill animal and excise tumor for storage at -80°C.
(TIF)

S2 Fig K^trans^ and AUC maps of all slices of the entire tumor of three animals. The first example is a mouse with complete tumor response at 2 weeks after PDT, while the other two had recurring growth, both in the distal part of the tumor. K^trans^ right after PDT and AUC at 72 h after PDT are shown. For K^trans^, only the contrast-enhanced pixels in the tumor are color coded. Both for example 2 and 3, high K^trans^ values and significant enhancement were seen in the distal part of the tumor (red arrows). In the entire tumor of example 1, and in the central and proximal tumor parts of examples 2 and 3, few pixels with high K^trans^ and AUC were observed.
(TIF)
