# Supplementary figures and images for: Quantitative Multi-Parametric Magnetic Resonance Imaging of Tumor Response to Photodynamic Therapy
Source: PLoS One. 2016 Nov 7;11(11):e0165759. doi: 10.1371/journal.pone.0165759 (PMC5098733; doi:10.1371/journal.pone.0165759)

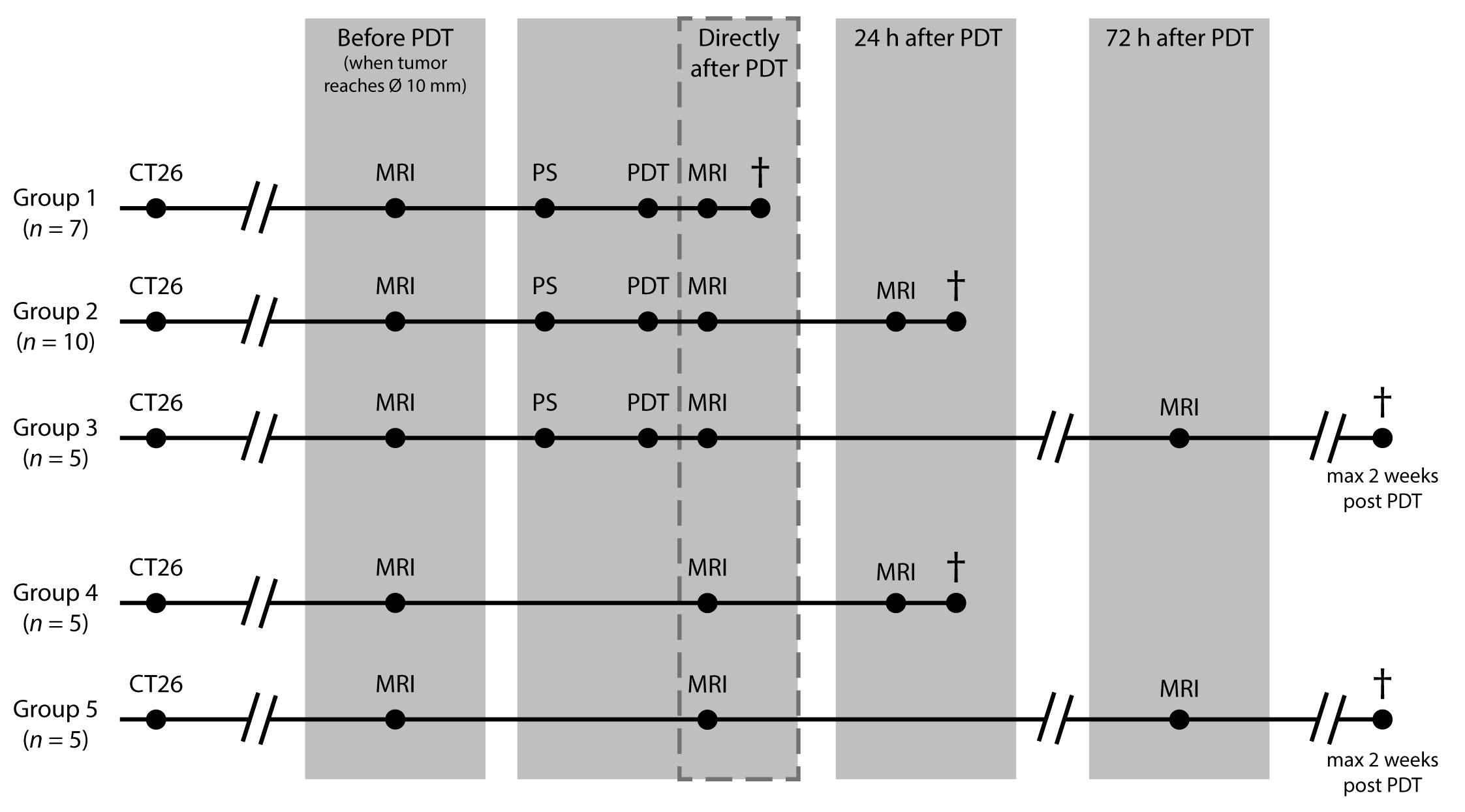

Supplement: S1 Fig — Numbers of animals are indicated per group. Grey blocks represent different measurement days. The following abbreviations are used: CT26: subcutaneous inoculation of CT26.WT tumor cells. MRI: Multi-parametric MRI scan session (approximate duration: 2.5 h). PS: photosensitizer injection, 6 h before PDT. PDT: photodynamic therapy (10 min tumor irradiation). †: kill animal and excise tumor for storage at -80°C. (TIF) [file pone.0165759.s001.tif]

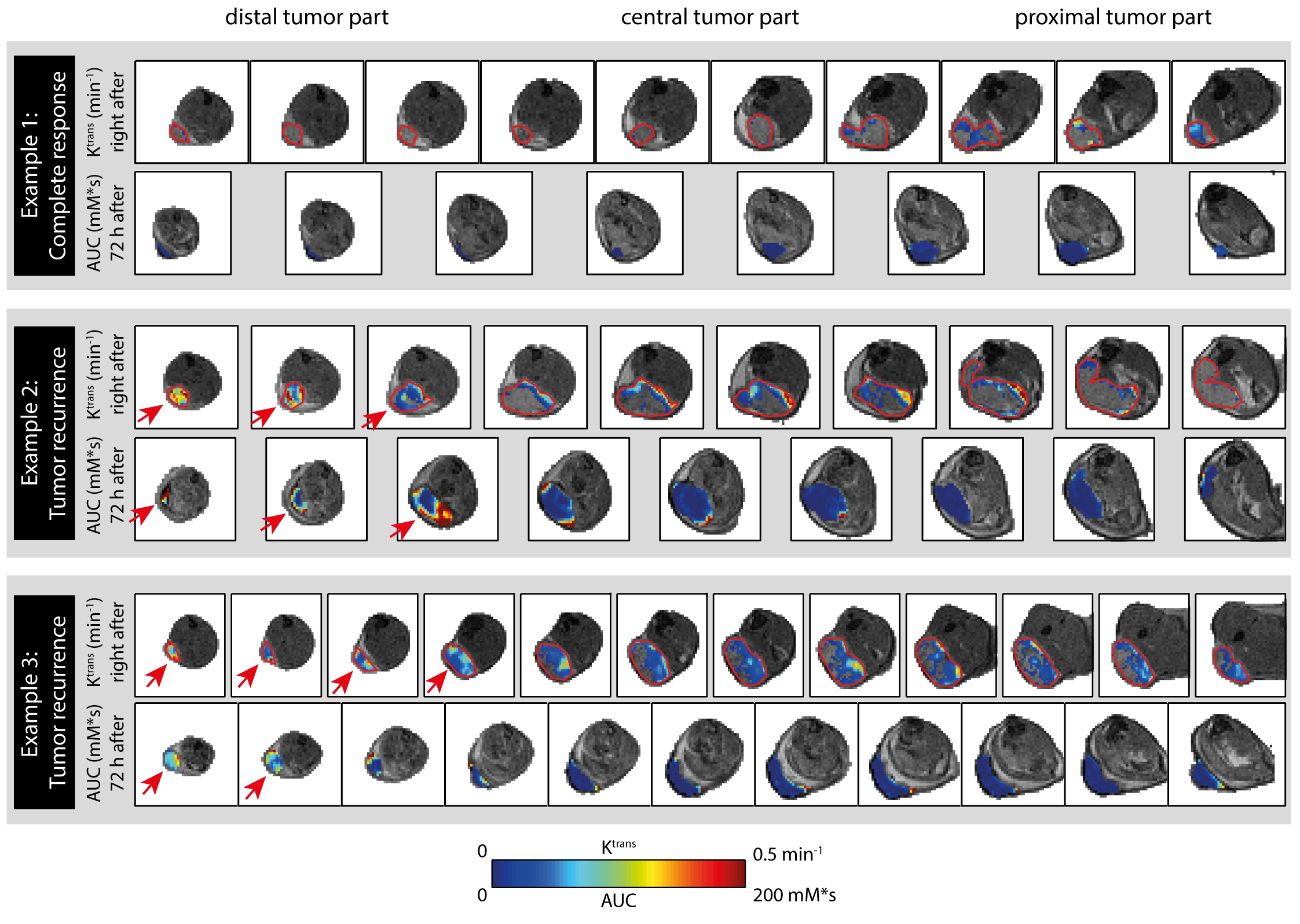

Supplement: S2 Fig — The first example is a mouse with complete tumor response at 2 weeks after PDT, while the other two had recurring growth, both in the distal part of the tumor. Ktrans right after PDT and AUC at 72 h after PDT are shown. For Ktrans, only the contrast-enhanced pixels in the tumor are color coded. Both for example 2 and 3, high Ktrans values and significant enhancement were seen in the distal part of the tumor (red arrows). In the entire tumor of example 1, and in the central and proximal tumor parts of examples 2 and 3, few pixels with high Ktrans and AUC were observed. (TIF) [file pone.0165759.s002.tif]
